# Vasculotide, an Angiopoietin-1 mimetic, ameliorates several features of experimental atopic dermatitis-like disease

**DOI:** 10.1186/s13104-015-1817-1

**Published:** 2016-05-28

**Authors:** Annie Bourdeau, Paul Van Slyke, Harold Kim, Maribelle Cruz, Tracy Smith, Daniel J. Dumont

**Affiliations:** Department of Immunology, University of Toronto, Toronto, ON USA; Sunnybrook Research Institute, Toronto, ON USA; Vasomune Therapeutics, 101 College Street, Toronto, ON USA; Department of Medical Biophysics, University of Toronto, Toronto, ON USA

**Keywords:** Tie2, Atopy, Eosinophil, Inflammation, Angiopoietin, Vasculotide, Psoriasis, Atopic dermatitis

## Abstract

**Background:**

Earlier studies by our group have demonstrated that a transgenic animal engineered to express Tie2 under the control of the Tie2 promoter produced animals with a scaly skin phenotype that recapitulated many of the hallmarks of atopic dermatitis (AT-Derm). To test the hypothesis that this model of AT-Derm is driven by dysregulated Tie2-signalling, we have bred AT-Derm transgenic (TG) animals with TG-animals engineered to overexpress Angiopoietin-1 or -2, the cognate Tie2 ligands. These two ligands act to antagonize one another in a context-dependent manner. To further evaluate the role of Ang1-driven-Tie2 signalling, we examined the ability of Vasculotide, an Ang1-mimetic, to modulate the AT-Derm phenotype.

**Results:**

AT-Derm+Ang2 animals exhibited an accentuated phenotype, whereas AT-Derm+Ang1 presented with a markedly reduced skin disease, similarly VT-treated AT-Derm animals present with a clear decrease in the skin phenotype. Moreover, a decrease in several important inflammatory cytokines and a decrease in the number of eosinophils was noted in VT-treated animals. Bone marrow differentiation in the presence of VT produced fewer CFU-G colonies, further supporting a role for Tie2-signalling in eosinophil development. Importantly, we demonstrate activation of Tie2, the VT-target, in lung tissue from naïve animals treated with increasing amounts of VT.

**Conclusions:**

The AT-Derm phenotype in these animals is driven through dysregulation of Tie2 receptor signalling and is augmented by supplemental Ang2-dependent stimulation. Overexpression of Ang1 or treatment with VT produced a similar amelioration of the phenotype supporting the contention that VT and Ang1 have a similar mechanism of action on the Tie2 receptor and can both counteract the signalling driven by Ang2. Our results also support a possible role for Tie2-signalling in the development of eosinophilic diseases and that activation of Tie2 may directly or indirectly modulate the differentiation of eosinophils, which express Tie2. In summary, these data support the hypothesis that this AT-Derm mouse model is driven by dysregulation of the Tie2 signalling pathway and increased Ang2 levels can aggravate it, whereas it can be reversed by either Ang1-overexpression or VT treatment. Moreover, our data supports the contention that VT acts as an Angiopoietin-1 mimetic and may provide a novel entry point for Tie2-agonist-based therapies for atopic diseases.

## Findings

### A Tie2-driven model of AT-Derm

**Fig. 1:**
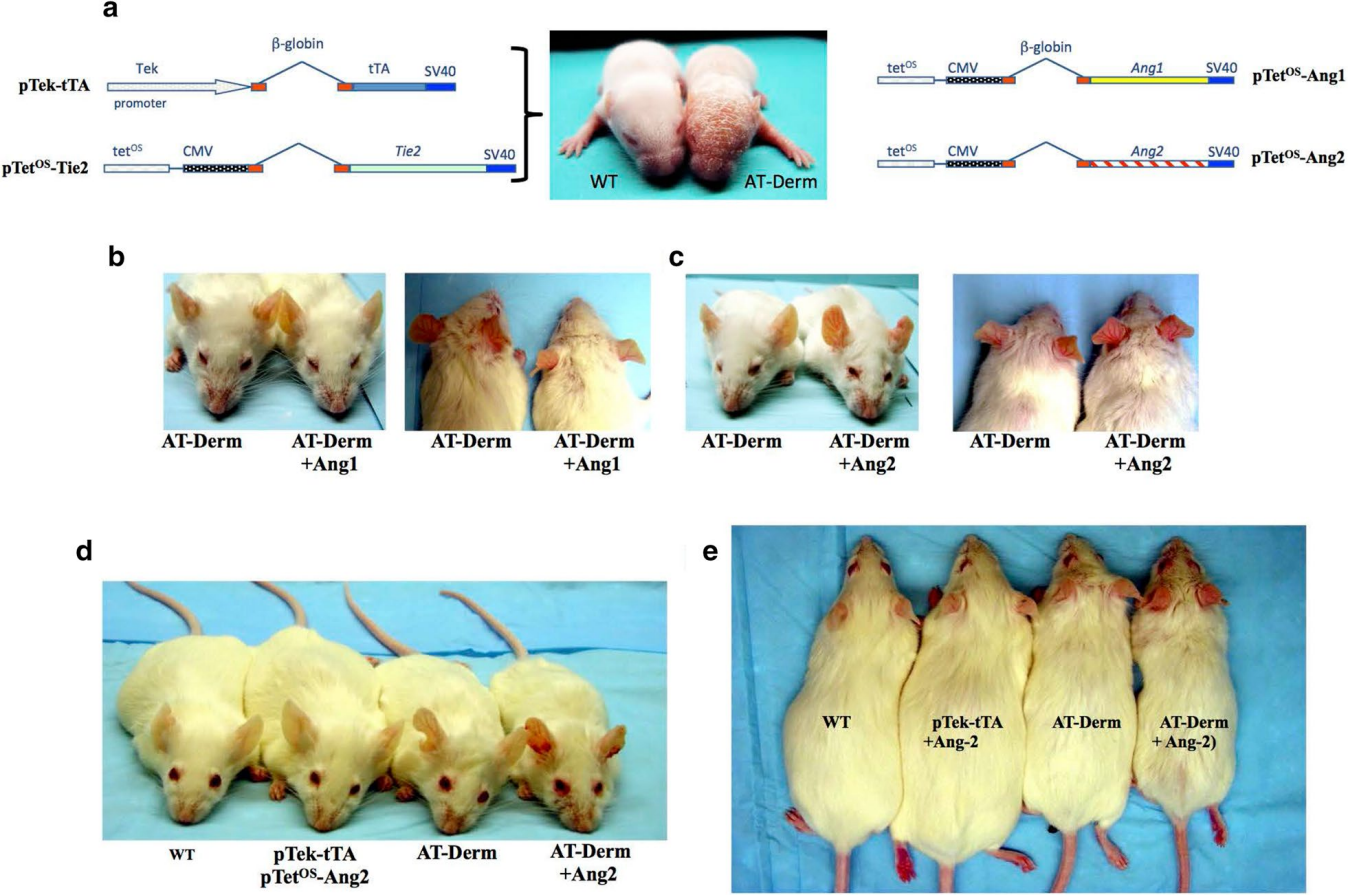
Depiction of the transgenes used in this study and the AT-Derm phenotype. **a** Diagram of the engineered transgenes. The animals expressing pTek-*tTA* and pTet^os^-*Tie2* together develop the AT-Derm phenotype as seen in 7 day old mice (*right*) compared to a wildtype littermate (*left*). The scaly skin phenotype of the AT-Derm animal is clearly evident. The Ang1 and Ang2 responder transgenes are also depicted to the right. **b** Gross phenotypic improvement is noted when experimental AT-Derm mice are crossed to the Ang1 pTet^OS^-*Ang1* mice, triple transgenic (pTek-*tTA*: pTet^OS^-*Tie2:* pTet^OS^-*Ang1*). Superficially, these effects are seen by a decrease in erythema around the nose and eyes. Also noted is a reduction in ear micro haemorrhages and tail scaly plaque formation (not shown). **c** AT-Derm Ang2-expressing triple transgenic mice present with increased erythema, micro haemorrhages and tail plaque (not shown). **d** Wild type and the Ang2-double transgenic (pTek-*tTA:* pTet^OS^-*Ang2*) exhibit no phenotype, whereas, the AT-Derm present with the characteristic erythema around the nose and eyes, which is much more accentuated in the AT-Derm+Ang2 triple transgenics. **e** Same animals as in *panel* **d** viewed from above, further illustrating the increased micro-hemorrhages in the ears

In two earlier publications, we describe the generation and characterization of a doxycycline-controlled transgenic mouse [1, 2] that presents with many of the physical hallmarks of psoriasis, however more extensive analysis of cytokine production and cellular infiltrate into the skin revealed that this model more accurately reflects AT-Derm [2]. Thus, this model provides an entry point into investigating some of the early factors that control the appearance and propagation of AT-Derm. In this study, we set out to test the hypothesis that this model of AT-Derm was driven by dysregulation of Tie2-signalling by either of the Tie2-cognate ligands, Ang1 or Ang2. In order to investigate the function of Ang1 and Ang2 in vivo, we engineered several transgenic lines that contained the Tet-responsive element upstream of either Ang1 or Ang2 (Fig. 1a) [3, 4]. These animals provided a way to regulate the overexpression of either of these ligands with doxycycline when mated with transgenic animals expressing the tetracycline-responsive transactivator, tTA. AT-Derm animals, which contain the two transgenes, pTek-tTA & tet^os^-Tie2 (Fig. 1a), that drive the AT-Derm phenotype, were bred to express a third transgene, pTet^os^-Ang1 and now present with an amelioration of the phenotype (Fig. 1b), whereas AT-Derm-Ang2 animals exhibit a much more severe phenotype. These AT-Derm-Ang2 animals present with increased erythema around the eyes, ears and snout and increased micro-hemorrhages in the ears (Fig. 1c). Importantly, the Ang2-accentuated phenotype is not observed in the absence of the Tie2-expressing transgene (tet^os^-Tie2) (Fig. 1d, e, pTek-tTA+Ang2), demonstrating that the AT-Derm phenotype depends upon on the expression of Tie2-transgene.

### VT-treatment resolves the AT-Derm phenotype

**Fig. 2:**
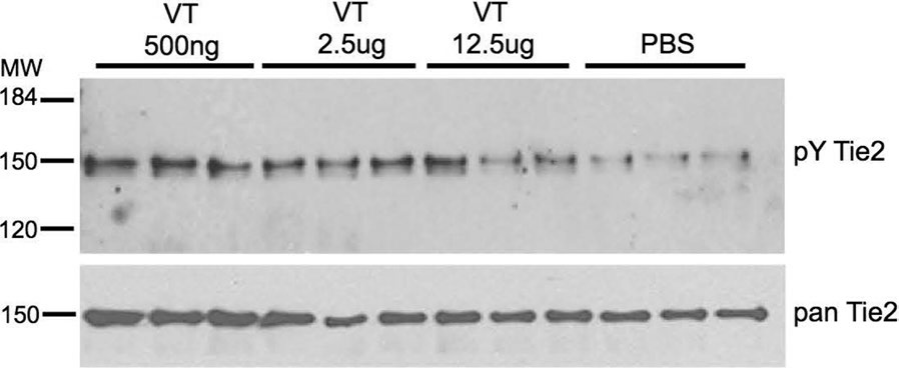
VT activates Tie2 in vivo. Lung tissue was removed from mice treated with different concentrations of VT for 1 h. Animals were sacrificed and lung tissue processed by immunoprecipitation for Tie2 and western blotted with anti-pY and anti-Tie2 antibodies

The fact that overexpression of Ang1 could reduce the severity of the AT-Derm phenotype provided the impetus for us to examine the possibility of substituting Ang1-overexpression with the Ang1-mimetic, Vasculotide. In order to demonstrate that VT engaged Tie2 and led to its activation we performed an acute short-term stimulation. In Fig. 2 we demonstrate that VT is able to activate Tie2, however this activation decreases as higher concentrations of VT are administered. The exact explanation for this decreased activation at higher concentrations of VT is not completely understood, but may reflect several properties of Tie2 signalling, one of which is the ability of VT to cluster a sufficient number of receptors on the cell surface and if VT is present in excess this ability is lost as we drive more monomeric binding of VT to Tie2. Also the mode of Tie2 activation is considered quite complex and may involve homotypic and heterotypic-interactions with other surface molecules [5, 6]. The requirement for Tie2 clustering by the Angiopoietins to drive activation has been demonstrated by several different groups using different methods of clustering Tie2. Such approaches, including COMP-Ang1 [7], COMP-Ang2 [8], MAT-Ang1 [9], Bow-Ang1 [10], all support the notion that the appropriate tetrameric or higher order multimeric clustering of Tie2 drives Tie2 signaling similar to that of parental ligand, Ang1. Further supporting a role for clustering of the Angiopoietins, we have demonstrated using a dominant-interfering form of Ang1, CC-Ang1, that both in vitro and in vivo the Angiopoietins need to be appropriately clustered to activated Tie2 [11]. Taken together the results presented here suggests that VT binds to Tie2 leading to its activation presumably through clustering of the receptor on the surface of Tie2-expressing cells.

**Fig. 3:**
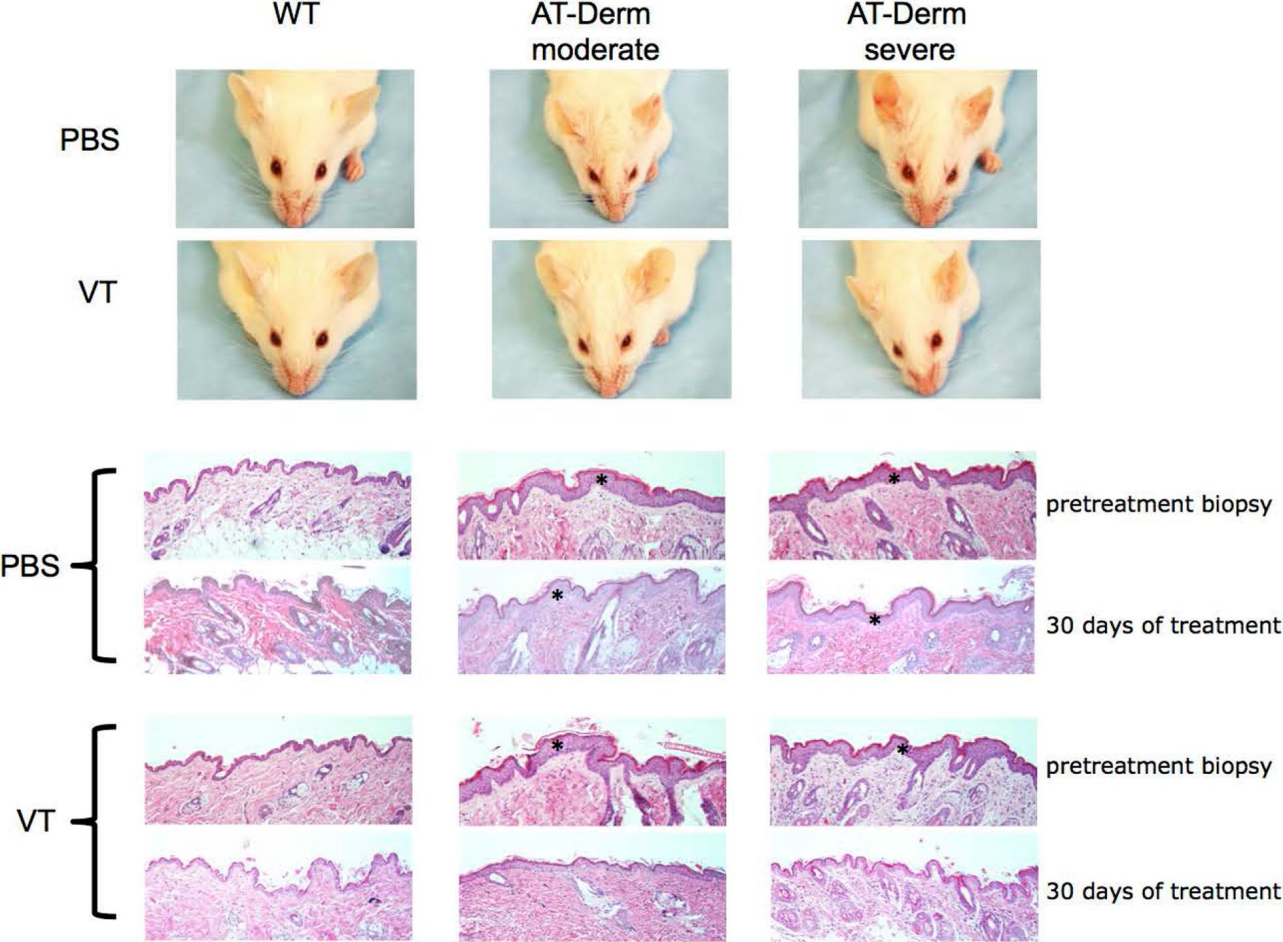
Treatment of AT-Derm animals with VT ameliorates the phenotype. Wildtype or AT-Derm animals with either moderate to severe phenotype were treated with VT at 40 µg/kg every 3 days for 30 days. Skin biopsies were taken from the back prior to initiation of treatment and at the conclusion of the treatment protocol. *Panels* correspond to the same animals post- and pre-treatment. Note the epidermal hyperplasia (*asterisk*) and immune cell infiltrate, which is unchanged in animals treated with PBS alone. In contrast, animals receiving VT-treatment presented with an epidermal thickness that resembled normal WT animals either treated with PBS or VT

The ability of VT to activate Tie2 in vivo (Fig. 2) [12–17] led us to investigate if VT was able to act like Ang1 in this model and impact the disease phenotype. As we have demonstrated in previous papers the AT-Derm animals have varying degrees of phenotype penetrance, thus they are classified as severe or moderate. Mice in each of these classes were grouped based on overt skin topology. These animals were treated with either PBS or VT for 30 days via intraperitoneal injection every third day. Prior to the initiation of treatment skin biopsies were taken and at the completion of the treatment regime a post-treatment biopsy was taken. Thin sections from these biopsies were stained with H&E. As first described in our earlier manuscripts AT-Derm animals carry many of the hallmarks of atopic dermatitis including epidermal hyperplasia, increased vascular density and immune cell infiltrates [1, 2]. This skin phenotype was not apparent in wild type animals, while PBS (vehicle) treated AT-Derm animals did present the skin phenotype. Significantly, the skin from VT-treated AT-Derm animals closely resembled the tissue taken from WT animals (Fig. 3). Thickening of the epidermis was no longer evident and there was a decrease in the vessel and immune cell density. The resolution of the AT-Derm skin phenotype mirrored that of AT-Derm-Ang1 animals supporting the notion that VT, like Ang1, is able to counteract the Ang2-Tie2 signaling axis and drive an Ang1-Tie2-like signaling pathway. Of additional interest is the fact that Ang1 is known to engage in signalling through alternate receptors [18, 19], however, since VT is only known to bind to Tie2 [12, 16] it suggests that virtually all of the AT-Derm phenotype is driven through Tie2-signalling and not through these alternate Ang-receptors.

### VT treatment leads to a decrease in the expression of pro-inflammatory molecules

**Fig. 4:**
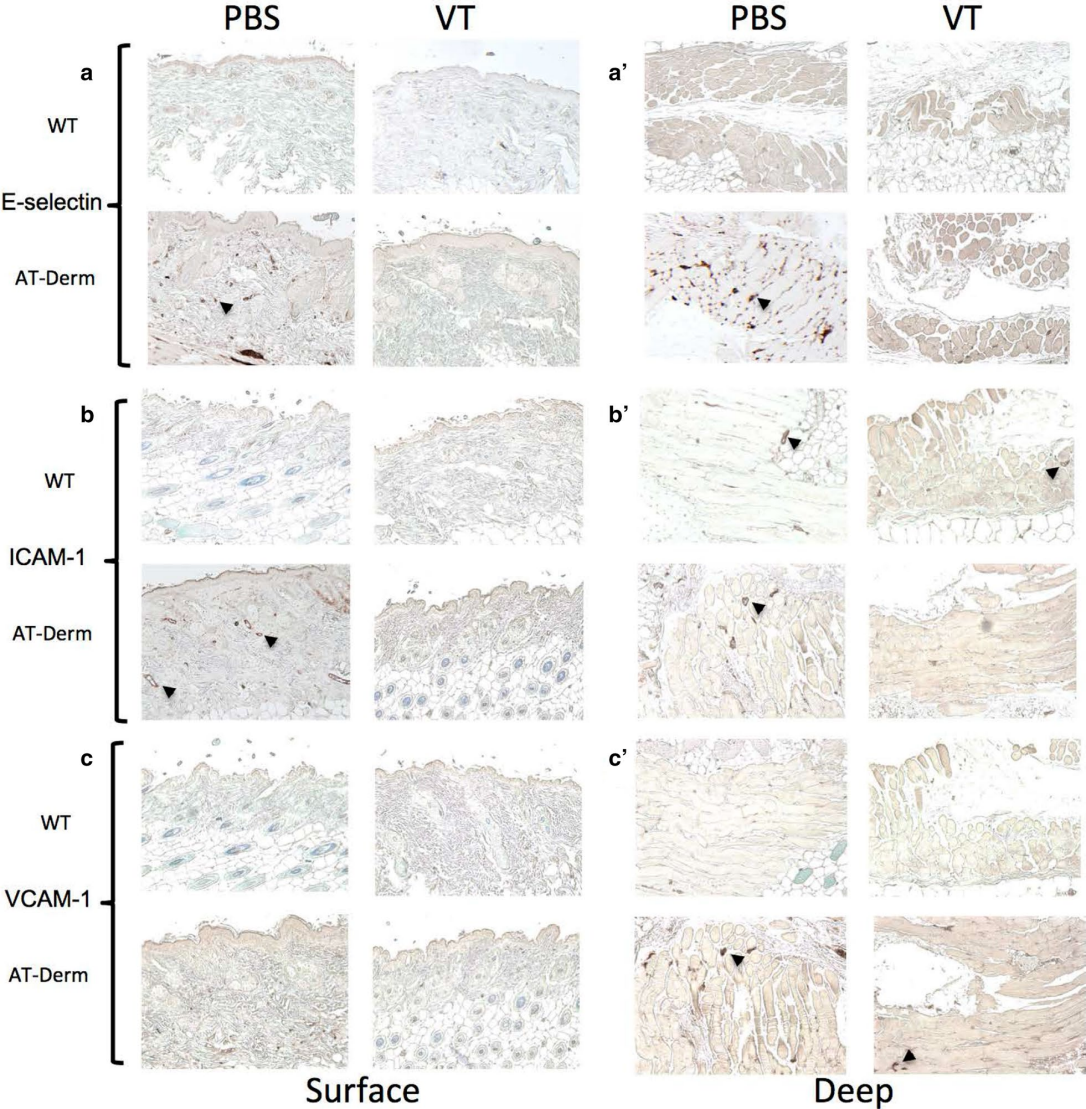
VT treatment decreases the expression of important inflammatory cell adhesion proteins. Thin sections prepared from WT and AT-Derm animals treated as described above with either PBS or VT were processed for immunohistochemical analysis for the presence of three cell surface proteins. **a**,**a’** E-selectin, **b**,**b’** ICAM-1 and **c**,**c’** VCAM-1. Expression of all of these proteins was not detectable or was very low in wt animals. However in AT-Derm animals all of these proteins were expressed (*arrowheads*) and this expression was decreased by VT treatment. VT was effective at decreasing the expression of these proteins in the underlying muscular tissue as well (**a’**, **b’**, **c’**)

Immunohistochemical analysis of thin sections taken from the animals described above treated with either PBS or VT demonstrated that VT treatment resulted in the down-regulation of E-selectin, ICAM-1 and VCAM-1 (Fig. 4a–c). Moreover, this down-regulation of cell adhesion receptor expression was detected in the underlying skeletal muscle layer below the affected skin (Fig. 4a–c).

**Fig. 5:**
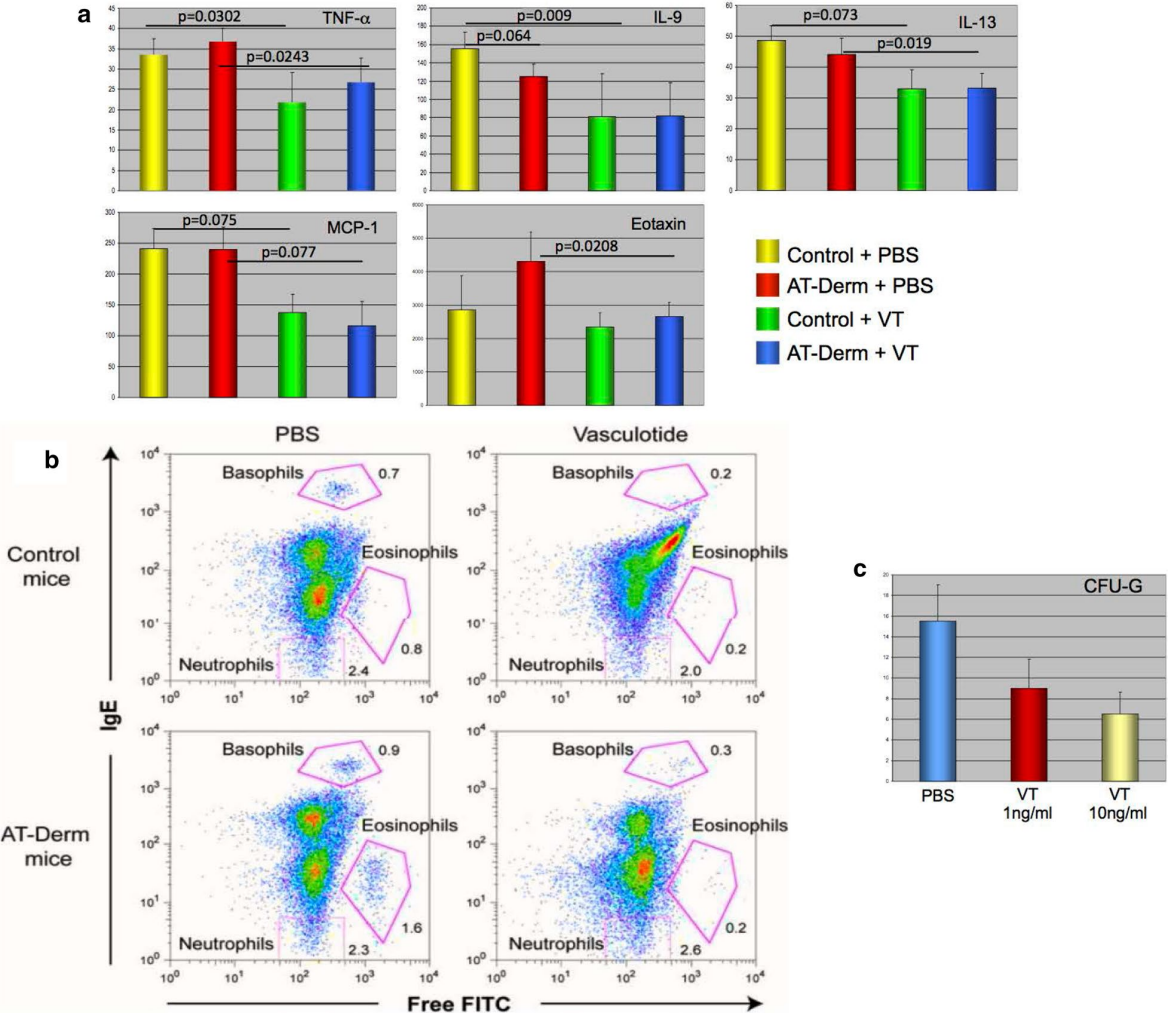
VT treatment decreases the expression of Eotaxin and other important inflammatory cytokines and decreases the number of eosinophils produced in vitro and in vivo. **a** Plasma from the animals treated as describe previously analyzed by either cytokine bead array for TNF-α, IL-9, IL-13 or MCP-1 or by ELISA for Eotaxin. p < 0.05 was considered statistically significant. Statistics were performed as pair-wise analysis with groups shown significant differences indicated. **b** Peripheral blood was drawn from animals treated with vehicle or VT for 30 days. Blood was processed for FACS analysis for surface IgE and FITC absorption. The decreased number of basophils and eosinophils in treated normal and AT-Derm mice is clearly evident in noted gates, while no effect was seen on neutrophils. **c** Dissociated bone marrow from CD1 mice was taken and plated in Methocult GF M3434 media for 7 days. Hematopoietic colonies were enumerated. A dose dependent, statistically significant reduction in CFU-G numbers was noted in response to VT treatment

The decrease in the expression of pro-inflammatory adhesion molecules led us to investigate the expression of key cytokines in the blood of treated animals. Several different leukocyte populations play a role in the early inflammatory response, including eosinophils. The expression of key inflammatory proteins involved in the early eosinophilic response (TNF-alpha, IL-9, IL-13, MCP-1 and Eotaxin) [20–22] in VT-treated animals was decreased in both WT and AT-Derm animals, suggesting that VT is able to modulate the inflammatory state both in normal and diseased animals (Fig. 5a).

The decrease in Eotaxin levels in VT-treated animals and the fact that eosinophils express Tie2 and respond to Angiopoietin 1 and 2 [23] guided us to examine the impact of VT on the number of eosinophils and their differentiation [20, 21]. Figure 5b illustrates that VT treatments leads to a decrease in the number of eosinophils and basophils in both AT-Derm and normal mice. These results imply that VT may either directly or indirectly impact the circulating numbers of eosinophils in the blood. Moreover, bone marrow extracted from mice and cultured in Methocult M3434 media demonstrated a VT-dose-dependent decrease in the number of CFU-G colonies detected, suggesting that the impact of VT on eosinophil numbers in circulation may depend on Tie2-activity in an early differentiation step.

The cellular target responsible for the noted VT-driven decrease in eosinophil number is very difficult to discern from these experiments. The obstacles in further defining the cell(s) responsible for this effect reside in the fact that HSC, eosinophils and ECs all express Tie2 and as such, likely all serve as targets for VT-driven activation of Tie2 [23, 24]. Thus, whether VT-driven Tie2 signalling is having a direct or indirect impact on this differentiation program has not been resolved. However, the fact that VT agonizes Tie2 illustrates that modulation of the Tie2 signaling pathway leads to down-regulation of the inflammatory cascade present in this model of AT Derm.

## Conclusions

In this manuscript we demonstrate that experimentally induced AT-Derm disease can be modulated through the Tie2 receptor with either Ang1-, Ang2- or VT-mediated signalling. The impact that VT-driven Tie2 signaling has on cytokine production, eosinophil differentiation and the expression of pro-inflammatory cell adhesion molecules on the endothelium illustrates that Tie2 signalling exerts its anti-inflammatory effects through multiple cellular pathways. Moreover, the fact that VT behaves much like Ang1 in these studies further supports the contention that these two Tie2 agonists display indistinguishable mechanisms of action in this experimental model of AT-Derm.

## Methods

### Animals

The transgenic animals used in this study have previously been described [1–4, 25, 26]. The housing and treatment of animals followed all institutional guidelines and was approved by the animal ethics board of SRI.

### Western analysis

Lung tissue lysates were processed for Western analysis as previously described [1], with anti-Tie2 and anti-pY (4G10) antibodies.

### Immunohistochemical analysis

Thin sections from tissue prepared as described [1] were stained for the presence of VCAM, ICAM, and E-selectin (Pharmagen) using the antibody staining kit from Vector and horseradish peroxidase.

### VT treatment

Vasculotide is synthesized as described [14, 15, 17]. To examine the activation of Tie2 by VT CD-1 mice were injected intraperitoneally with indicated amounts of VT as described in the figure legend. One hour post VT-treatment animals were sacrificed and lung tissue removed for analysis. For AT-Derm studies animals were treated with VT at 40 ug/kg every third day for 30 days.

### Flow cytometry analysis

Cells from the peripheral blood were processed for the presence of eosinophils as described by the vendor BD. Blood was processed for FACS analysis for surface IgE and FITC absorption.

### Cytometric bead array analysis

CBA beads were purchased from BD that were directed against TNF-a, IL13, IL9 and MCP-1 and processed as described by the vendor. The eotaxin analysis was performed with an eotaxin-specific ELISA as per the vendors protocol (BD). Statistically significant differences are indicated (unpaired student *t* test, where p < 0.05).

### Bone marrow culture

Bone marrow was removed from the femur of normal CD-1 mice and processed in Methocult as described by the vendor (Stemcell Technologies).

## Abbreviations

Ang1 & 2: Angiopoietin-1, -2
COMP-Ang1 & COMP-Ang2: Cartilage oligomeric matrix protein
VT: Vasculotide
PEG: Polyethylene glycol
